# Improving associative memory in younger and older adults with unitization: evidence from meta-analysis and behavioral studies

**DOI:** 10.3389/fnagi.2024.1389957

**Published:** 2024-05-23

**Authors:** Zejun Liu, Yujuan Wang, Yajun Zhu, Jing Yuan, Wei Liu

**Affiliations:** ^1^Department of Psychology, Shanghai Normal University, Shanghai, China; ^2^Lab for Educational Big Data and Policymaking, Ministry of Education, Shanghai Normal University, Shanghai, China; ^3^Intellectual Property School, Chongqing University of Technology, Chongqing, China; ^4^Kangbai Hospital, Hangzhou, China; ^5^College of Nursing, Hebei University, Baoding, China

**Keywords:** unitization, associative memory, changes in the level of unitization, age groups, meta-analysis

## Abstract

**Introduction:**

The finding that familiarity can support associative memory by unitizing the to -be-learned items into a novel representation has been widely accepted, but its effects on overall performance of associative memory and recollection are still controversial.

**Methods:**

The current study aims to elucidate these discrepancies by identifying potential moderating factors through a combined approach of meta-analysis and behavioral experiment.

**Results:**

Results consistently showed that changes in the level of unitization and age groups were two important moderators. Specifically, unitization enhanced younger and older adults’ associative memory and its supporting processes (i.e., familiarity and recollection) when the level of unitization between studied and rearranged pairs was changed. However, when this level remained constant, unitization exhibited no impact on associative memory and familiarity in younger adults, but showed an enhanced effect in older adults. Furthermore, results revealed a marked group difference between younger and older adults in associative memory when the unitization level of noncompound words remained unaltered. Upon breaking this condition, the group difference was reduced by enhancing familiarity or recollection.

**Discussion:**

These findings not only clarify some of the inconsistencies in the literature concerning the impact of unitization on associative memory, but also suggest that unitization is a beneficial strategy for reducing group difference in associative memory, with its effectiveness varying according to the level of unitization changes.

## Introduction

A central feature of human memory is the ability to quickly form new connections between two unrelated pieces of information (Eichenbaum, 2003; Davachi, 2006). Indeed, this ability is a central mechanism driving our daily access to information, such as locating misplaced keys, recalling names, or determining the cost of an apple. It necessitates the retention of not only the individual items (e.g., location, keys, names, apple, i.e., item memory) but also the associations between these items (e.g., location—keys, people—names, cost—apple, i.e., associative memory) (Buchler et al., 2008; Bader et al., 2010). Dual-process theories posit that memory is based on two distinct processes: Familiarity and recollection. Familiarity is a sensation of having previously encountered items without the retrieval of supplementary details, while recollection means the controlled retrieval of additional contextual information pertaining to studied episodes (Yonelinas, 2002; Yonelinas et al., 2010).

Traditional perspectives argue that item memory is supported by both familiarity and recollection, while associative memory is solely supported by recollection. Recent research, however, has challenged this view by demonstrating that familiarity can also support associative memory when the to-be-learned items are unitized into a new representation. This manipulation of binding two or more unrelated items into a new representation is defined as unitization (Graf and Schacter, 1989; Yonelinas, 2002). Early researchers believed that unitization was “all or nothing,” meaning that there was either unitization or non-unitization (Bader et al., 2010; Pilgrim et al., 2012; Kamp et al., 2016). However, as the research progressed, Parks and Yonelinas (2015) used the term levels of unitization to refer to the idea that there is a continuum along which associations can be unitized. At the lower end of the continuum, two items may be treated as two separate objects, and the only way in which they are associated is that they have occurred in the same episodic context. At the higher end of the continuum, the two items may not even be perceived as two separate items at all but rather are processed as a single coherent entity or object. They also doubt that either extreme exists in a pure form and therefore refer to higher and lower unitized associations or high- and low-unitization conditions. The construct, as such, is a relative one, in the sense that there is no absolute level of unitization. Rather, conditions can be contrasted that vary in the degree to which the components of the association are treated a single or separate units (e.g., as two separate words vs. as a related word pairs or a compound words). Over the past two decades, a wealth of behavioral studies (Ahmad and Hockley, 2014; Ahmad et al., 2015; Delhaye et al., 2018, 2019; Delhaye and Bastin, 2018) and event-related potential (ERP) studies (Bader et al., 2010; Tibon et al., 2014; Zheng et al., 2015a,b; Kamp et al., 2016; Li et al., 2017, 2019; Han et al., 2018, 2022; Liu et al., 2022; Liu and Guo, 2022), employing various manipulation, age groups, and stimulus materials, have investigated the impact of unitization on associative memory (see Table 1). In the following section, we will provide a systematic review of these studies.

**Table 1:** Summary of study characteristics.

Study (year)	Exp name	Sample size	Manipulations of unitization	Age groups	Stimulus types	Language types	Changes in the level of unitization	Key data for associative recognition	Key data for familiarity	Key data for recollection
Ahmad and Hockley (2014)	Exp 1	30	CW vs. NCW	Younger	Word stimuli	English	No-change	*F* = 1.13	--	--
	Exp 2	22 vs. 22	CW vs. NCW	Younger	Word stimuli	English	No-change	2.10(1.0) vs. 2.12(1.2)	--	--
	Exp 3a	26	CW vs. NCW	Younger	Word stimuli	English	No-change	*t* = 2.5	--	--
							Change	*t* = 3.7	--	--
	Exp 3b	30	CW vs. NCW	Younger	Word stimuli	English	No-change	*t* = 3.6	--	--
							Change	*t* = 2.97	--	--
	Exp 4	30	CW vs. NCW	Younger	Word stimuli	English	No-change	*t* = −0.096	--	--
Ahmad and Hockley (2017)	Exp 1a	28	CW vs. NCW	Younger	Word stimuli	English	No-change	*t* = 0.70	--	--
	Exp 1b	24	CW vs. NCW	Younger	Word stimuli	English	No-change	*F* = 0.11	--	--
Ahmad et al. (2015)	Exp 1	24	CW vs. NCW	Younger	Word stimuli	English	No-change	*t* = 0.623	--	--
		24		Older				*t* = 4.1	--	--
	Exp2	24	CW vs. NCW	Younger	Word stimuli	English	No-change	*t* = 0.59	--	--
	Exp3	40	CW vs. NCW	Younger and older	Word stimuli	English	Change and no-change	*F* = 35.3	--	--
Bridger et al. (2017)		19	Related vs. unrelated pairs	Younger	Picture stimuli	--	No-change	---	*t* = 3.20	*t* = 3.66
		27		Older				---	*t* = 2.14	*t* = 2.16
Delhaye and Bastin (2018)	Exp 1	20	CW vs. NCW	Younger	Word stimuli	English	No-change	*t* = −0.45	*t* = 5.34	*t* = −1.55
				Older				*t* = 0.42	*t* = 2.39	*t* = 0
	Exp 2	20	CW vs. NCW	Younger	Word stimuli	English	No-change	*t* = 1.42	*t* = 2.89	*t* = −1.06
				Older				*t* = 2.91	*t* = 3.15	*t* = 1.34
Delhaye et al. (2018)		20	Related vs. unrelated pairs	Younger	Picture stimuli	--	Change	*t* = −0.52	*t* = 3.08	*t* = 0
				Older		--		*t* = −0.20	*t* = −0.95	*t* = −0.58
Delhaye et al. (2019)		34	Related vs. unrelated pairs	Younger	Word stimuli	English	Change	*t* = 3.92		
							No-change	*t* = 2.45		
				Older			Change	*t* = 7.83		
							No-change	*t* = 1.55		
Desaunay et al. (2017)		20	Related vs. unrelated pairs	Younger	Picture stimuli	--	No-change	*t* = 2.87	*t* = 2.17	*t* = 2.72
Ford et al. (2010)		18	CW vs. NCW	Younger	Word stimuli	English	No-change	*t* = −0.53	--	--
Greve et al. (2007)		15	Related vs. unrelated pairs	Younger	Word stimuli	English	No-change	*t* = 4.68	*t* = 2.45	1.87(3.95) vs. 1.44(3.95)
Gutchess and Park (2009)		68	Related vs. unrelated pairs	Younger and older	Picture stimuli	--	Change and no-change	*t* = 2.18	--	--
Han et al. (2022)	Exp 1	42	Related vs. unrelated pairs	Younger	Picture stimuli	--	No-change	*t* = 10.95	*t* = 2.07	*t* = 2.57
	Exp 2	40	Related vs. unrelated pairs	Younger	Picture stimuli	--	No-change	*t* = 14.86	*t* = 2.38	*t* = 3.30
Huffer et al. (2022)		46	Related vs. unrelated pairs	Younger and older	Picture stimuli	--	No-change	*t* = 5.38	--	--
Jin (2021)		19	CW vs. NCW	Younger	Word stimuli	Chinese	Change	*t* = −1.33	--	--
		23		Older				*t* = 2.25	--	--
Kriukova (2010)	Exp 2	20	Related vs. unrelated pairs	Younger	Word stimuli	English	Change	---	*t* = 2.25	*t* = 0.53
	Exp 4	20	Related vs. unrelated pairs	Younger	Word stimuli	English	Change	---	*t* = 3.12	*t* = 0.29
Kriukova et al. (2013)		16	Related vs. unrelated pairs	Younger	Word stimuli	Chinese	Change	*t* = 4.06	*t* = 0.88	*t* = 2.24
Li et al. (2017)		16	CW vs. NCW	Younger	Word stimuli	Chinese	Change	---	*F* = 4.45	*F* = 1.25
Li et al. (2019)		17	CW vs. NCW	Younger	Word stimuli	Chinese	No-change	*F* = 12.35	*F* = 5.05	*F* = 12.16
Li et al. (2021)		25	CW vs. NCW	Younger	Word stimuli	Chinese	No-change	*t* = 3.26	*t* = 2.09	*t* = 1.17
Liu et al. (2019)		18	CW vs. NCW	Younger	Word stimuli	Chinese	Change	*t* = 3.59	*t* = 3.61	*t* = 4.28
Liu et al. (2022)		29	CW vs. NCW	Younger	Word stimuli	Chinese	No-change	*t* = 5.67	*t* = 1.76	*t* = 1.82
Liu et al. (2020a)		33	CW vs. NCW	Younger	Word stimuli	Chinese	Change	*F* = 6.95	*F* = 6.29	*F* = 3.00
							No-change	*F* = 0.60	*F* = 1.18	*F* = 2.08
Liu et al. (2021a)	Exp 1a	17	CW vs. NCW	Younger	Word stimuli	Chinese	Change	*t* = 5.41	*t* = 3.79	*t* = 6.14
	Exp 1b	17	CW vs. NCW	Younger	Word stimuli	Chinese	Change	*t* = 5.50	*t* = 2.32	*t* = 3.40
Liu et al. (2020b)	Exp 1	33	CW vs. NCW	Younger	Word stimuli	Chinese	Change	*t* = 4.28	*t* = 3.56	*t* = 3.91
	Exp 2	23	CW vs. NCW	Younger	Word stimuli	Chinese	Change	*t* = 3.03	--	--
Liu et al. (2021b)		35	Related vs. unrelated pairs	Younger	Picture stimuli	--	Change	*F* = 94.10	*F* = 34.48	*F* = 0.44
Liu et al. (2022)		30	CW vs. NCW	Younger	Word stimuli	Chinese	Change	*t* = 8.45	*F* = 9.19	*F* = 13.56
							No-change	*t* = −0.93	*F* = 1.62	*F* = 1.35
Lv et al. (2015)		15	Related vs. unrelated pairs	Younger	Picture stimuli	--	Change	*t* = 6.06	*t* = 2.90	*t* = 3.78
Lv et al. (2017)		24	Related vs. unrelated pairs	Younger	Word stimuli	Chinese	Change	*t* = 4.57	*t* = 1.13	*t* = 2.27
Ma et al. (2021)		27	Related vs. unrelated pairs	Younger	Picture stimuli	--	Change	*t* = 11.12	*F* = 14.86	*F* = 15.47
						--	No-change	*t* = 5.41	*F* = 0.75	*F* = 0.16
Memel and Ryan (2017)		18	Related vs. unrelated pairs	Younger	Picture stimuli	--	No-change	*t* = 3.02	--	--
		24		Older		--		*t* = 7.10	--	--
Naveh-Benjamin (2000)		72	Related vs. unrelated pairs	Younger and older	Word stimuli	English	No-change	*t* = 7.00	--	--
Naveh-Benjamin et al. (2003)		60	Related vs. unrelated pairs	Younger and older	Word stimuli	English	No-change	*t* = 3.57	--	--
Ngo and Lloyd (2016)	Exp 1a	41	Related vs. unrelated pairs	Younger	Picture stimuli	--	No-change	*t* = 4.79	--	--
	Exp 1b	39	Related vs. unrelated pairs	Younger	Picture stimuli	--	No-change	*t* = 0.60	--	--
Patterson et al. (2009)		28 vs. 28	Related vs. unrelated pairs	Older	Word stimuli	English	No-change	0.26(0.21) vs. 0.20(0.18)	--	--
Tibon et al. (2014)		32	Related vs. unrelated pairs	Younger	Picture stimuli	--	Change	*t* = 5.76	*F* = 9.75	*F* = 8.23
Wang et al. (2016)		20	Related vs. unrelated pairs	Younger	Word stimuli	Chinese	Change	*t* = 9.70	*t* = 1.97	*t* = 0.88
Wang and Li et al. (2021)		20	CW vs. NCW	Younger	Word stimuli	Chinese	Change	*t* = 0	--	--
		20		Older				*t* = −0.41	--	--
Zheng et al. (2015a)		24	CW vs. NCW	Younger	Word stimuli	Chinese	Change	*t* = 1.01	--	*t* = 0.56
				Older				*t* = 3.70	--	*t* = 2.65
Zheng et al. (2015b)		20	CW vs. NCW	Younger	Word stimuli	Chinese	Change	*F* = 6.64	*F* = 5.23	*F* = 7.39

CW means compound words; NCW means noncompound words.

In the column of study (year), a light-colored row (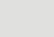, 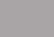) indicates the inclusion of a single experiment in the study, whereas a dark-colored row (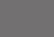) indicates the incorporation of multiple experiments. These information can also be found in the column of experimental name, with a null value indicating the presence of only one experiment in the study. In the last two columns, “*--*” indicates that the studies only focused on the impact of unitization on associative memory, but not on familiarity and recollection. In the third penultimate column, “*---*” means missing values, indicating that the data in those cells is not accessible or available. In the last three columns, there are three types of original effect sizes extracted from each studies: *t*-value, *F*-value and mean (SD). These key data could be used to calculate the effect size in this study, as described in the section of effect size calculation on page 17.

## Effect of unitization on younger adults

Numerous behavioral and ERP studies have investigated the impact of unitization on associative memory and its supporting processes (see Table 1). Compound words and related pairs are the most commonly used ways to manipulate the level of unitization. Some studies manipulating level of unitization with compound vs. noncompound words revealed that unitization may either enhance or not affect associative memory (facilitate: Li et al., 2017, 2019, 2021; Liu et al., 2020a,b, 2021b; not facilitate: Ford et al., 2010; Ahmad and Hockley, 2017; Delhaye and Bastin, 2018; Delhaye et al., 2018). When examining its effects on the two processes, familiarity and recollection, most of these studies agreed that unitization could promote familiarity to support associative memory (Li et al., 2017, 2019; Delhaye and Bastin, 2018; Delhaye et al., 2018; Liu et al., 2020a,b, 2021b), but they reported mixed findings regarding its impact on recollection, either an enhanced effect (Zheng et al., 2015b; Li et al., 2019; Liu et al., 2021b) or no effect (Li et al., 2017, 2021; Delhaye and Bastin, 2018; Delhaye et al., 2018). Furthermore, 22 studies manipulating level of unitization with related vs. unrelated stimuli pairs, with 13 studies using picture pairs and nine using word pairs, reported an enhanced effect on familiarity, but the findings for its impact on associative memory and recollection have been inconsistent and varied considerably across studies (enhanced effect: Tibon et al., 2014; Lv et al., 2015; Bridger et al., 2017; Ma et al., 2021; Han et al., 2022; no effect: Desaunay et al., 2017; Delhaye and Bastin, 2018; Delhaye et al., 2018, see Table 1).

## Effect of unitization on older adults

Cognitive decline associated with aging results in older adults exhibiting a significant reduction in associative memory compared to younger adults, a phenomenon referred to as the age-related deficit in associative memory (Cohn et al., 2008; Badham et al., 2012; Bastin et al., 2013; Bridger et al., 2017; Liu and Liu, 2022). This well-known age-related deficit is thought to be due to two reasons. One is the difficulty in binding together the components of an association (Cohn et al., 2008; Delhaye and Bastin, 2018), and the other is the impaired recollection in older adults, contrasted with relatively preserved familiarity (see Davidson and Glisky, 2002 for a review). The enhanced effect of unitization on familiarity in younger adults encourages researchers. They speculate whether unitization can also improve associative memory in older adults by increasing reliance on familiarity and decreasing reliance on recollection. Some studies demonstrated this facilitation effect (Naveh-Benjamin et al., 2003; Zheng et al., 2015a; Bridger et al., 2017; Delhaye et al., 2019), while others did not (Delhaye et al., 2018; Delhaye and Bastin, 2018; Jin, 2021; Wang and Li, 2021; Huffer et al., 2022). When distinguishing the contribution of familiarity and recollection, Delhaye and its colleagues showed no effect of unitization on either familiarity or recollection (Delhaye et al., 2018; Delhaye and Bastin, 2018), while Zheng et al. (2015a) revealed an enhanced familiarity-related FN400 effect (i.e., early frontal old/new effect) but an equivalent recollection-related LPC^1^ effect (i.e., late left-parietal effect) under high-unitization conditions.

## Age-related deficit in unitization

In addition to examining the effect of unitization on associative memory in younger and older adults separately, a few researchers have also compared the group difference (i.e., the age-related deficit mentioned earlier) under different unitization conditions (Ahmad et al., 2015; Memel and Ryan, 2017; Delhaye et al., 2018, 2019; Delhaye and Bastin, 2018; Huffer et al., 2022). The existing studies that manipulated the level of unitization with compound vs. noncompound words (i.e., high- vs. Low-unitization conditions) or related vs. unrelated pairs (i.e., high- vs. Low-unitization conditions) have identified three patterns of results. One pattern revealed that under high unitization conditions, older adults showed associative memory equivalent to or better than younger adults, while under low unitization conditions, older adults exhibited lower performance of associative memory than younger adults (Naveh-Benjamin, 2000; Patterson et al., 2009; Ahmad et al., 2015). Another pattern showed that younger adults consistently outperformed older adults under both high and low unitization conditions, but the group difference was more pronounced under low unitization conditions (Naveh-Benjamin et al., 2003; Zheng et al., 2015a; Bridger et al., 2017; Delhaye et al., 2019). Both the two patterns of result suggest that unitization does reduce group difference. And beyond that, the third pattern failed to observe such reduction, with younger adults exhibiting higher performance of associative memory under both high and low unitization conditions, and no significant group difference between the two conditions (Memel and Ryan, 2017; Delhaye et al., 2018; Delhaye and Bastin, 2018; Huffer et al., 2022). When examining the contributions of familiarity and recollection to this group difference, the results also show variability. Delhaye and its colleagues found that younger adults showed enhanced recollection for noncompound words compared to older adults, while in recollection for compound words and in familiarity for both compound and noncompound words, there were no significant group difference (Delhaye and Bastin, 2018). Additionally, they also found that younger adults showed enhanced familiarity than older adults for both related and unrelated picture pairs, but there was no significant group difference in recollection (Delhaye et al., 2018). In contrast, Zheng et al. (2015a) found that younger adults elicited larger familiarity-related FN400 and recollection-related LPC effects for noncompound words compared to older adults, while compound words elicited considerable FN400 and LPC effects between the two age groups.

Collectively, these studies indicate that unitization consistently promotes familiarity to support associative memory for both younger and older adults, but its effects on associative memory and recollection remain controversial. Why do these results differ? What potential factors may moderate the effects of unitization on associative memory and recollection?

## Potential moderators of unitization effect

In addition to the obvious differences in manipulations of unitization, age groups, stimulus types and language types, we have found a clear divergence across these studies, namely, changes in the level of unitization between studied and rearranged pairs (Liu et al., 2022). Specifically, participants learned compound words (high-unitization condition) and noncompound words (low-unitization condition) during encoding. Then, these compound words might be rearranged into other compound words or noncompound words during retrieval, while these noncompound words were rearranged into other noncompound words. In the former case, the level of unitization between studied and rearranged pairs may or may not change, whereas in the latter case, the level of unitization does not change. Checking the studies mentioned above, nearly 40% of the studies did not consider this factor (see the column of “changes in the level of unitization” in Table 1). A bold speculation is that changes in the level of unitization between studied and rearranged pairs might be another important factor that can moderate the effects of unitization on associative memory. Experimental evidences supporting this speculation were shown by Delhaye et al. (2019), Liu et al. (2020a, 2022). These three studies consistently found that, compared to the no impact observed under no-change conditions, unitization exhibited an enhanced effect under change conditions for both younger and older adults.

## Purposes of the current study

Therefore, the purpose of this study is to investigate possible moderating factors that can explain the different effects of unitization on associative memory and the supporting processes involved, with the use of meta-analysis. Specifically, (1) How do different manipulations of unitization (e.g., compound vs. noncompound words, related vs. unrelated pairs) affect associative memory and its processes? (2) Do age groups (e.g., younger vs. older adults) influence the effects of unitization on associative memory and its processes? (3) Do changes in the level of unitization (e.g., change, no-change) moderate the effects of unitization on associative memory and its processes?

## Meta-analysis study

### Methods

#### Transparency and openness

We describe all literature search and selection, codes, and calculation of effect size in the study. All data are available from https://osf.io/sp9uv/files/osfstorage. Data were analyzed using Comprehensive Meta-Analysis (CMA 3.0) program. And because all data analysis is done through interface click operations, the analytic code was not available in this study. In addition, this study’s design and its analysis were not preregistered.

#### Literature search

We conducted a systematic search for peer-reviewed journal articles and dissertation reports using the following online databases: Web of Science, PsycINFO, Google Scholar, PubMed, and CNKI. The search was limited to papers published on or prior to August 31, 2022 and included the following search phrases: (“Unitization” OR “unit” OR “relatedness” OR “related” OR “compound”) AND (“associative memory” OR “associative recognition” OR “familiarity” OR “recollection” OR “FN400” OR “LPC”). Initially, a total of 1,404 papers were searched.

#### Study selection

To be included in this meta-analysis, studies had to meet the following criteria: (a) the sample included healthy participants (i.e., younger and older adults), studies involving individuals with amnesia, Parkinson’s disease, Alzheimer’s disease, and selective hippocampal damage were excluded; (b) the level of unitization must be manipulated using compound vs. noncompound words or related vs. unrelated pairs, as studies that manipulated level of unitization with other strategies were excluded^2^; and finally, (c) only the journal article was included when the dissertation and journal article used the same data.

**Figure 1:**
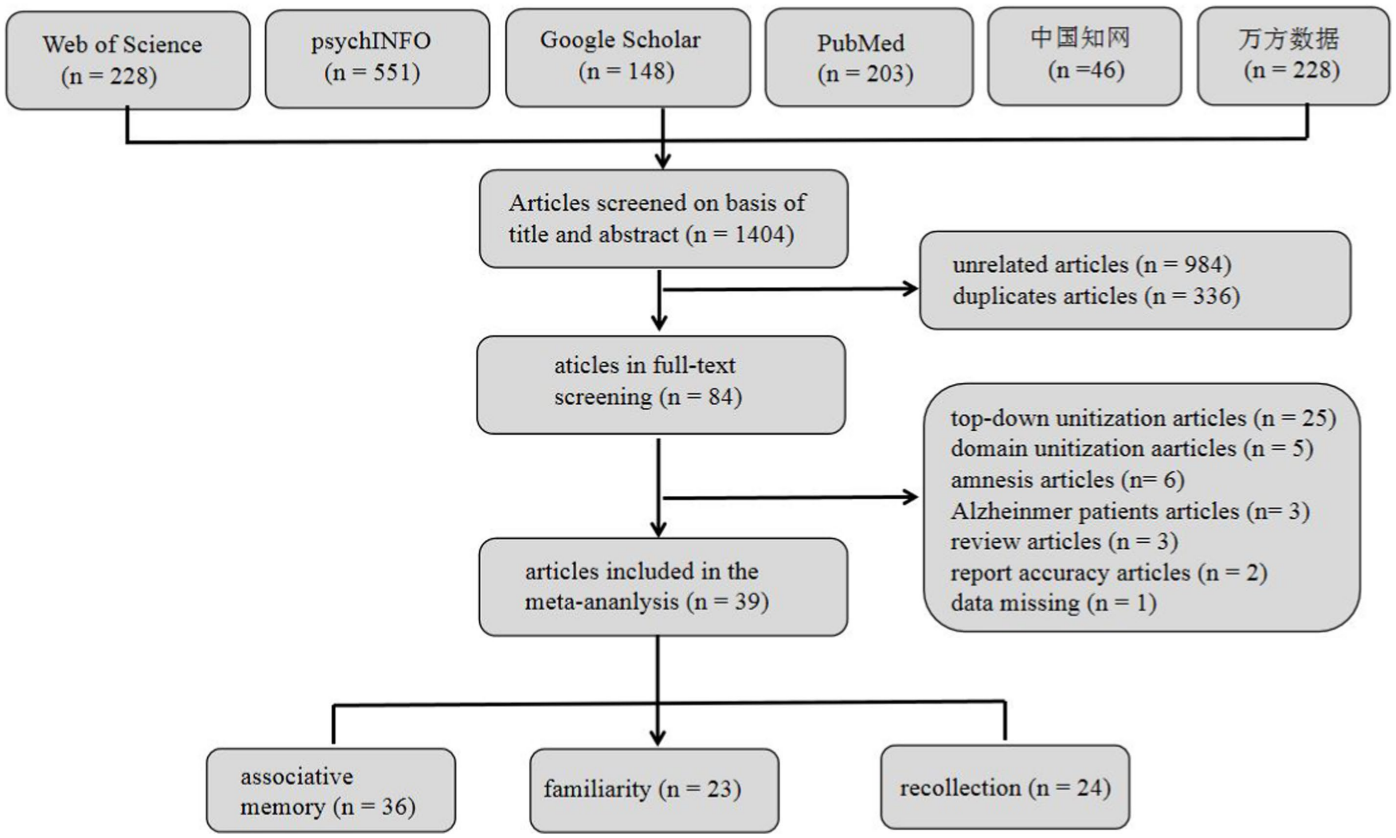
Flowchart of the study selection.

Figure 1 presents a flowchart of the study selection process, and Table 1 provides descriptions of these included studies. Initially, 1,404 articles were searched, and after excluding 984 unrelated and 336 duplicate articles, 84 articles remained. Full-text screening resulted in the inclusion of 39 studies that met the criteria for inclusion. Among these, 36 studies (63 study samples) examined the effect of unitization on associative memory, 23 studies (35 study samples) investigated its effect on familiarity, and 24 studies (36 study samples) explored its effect on recollection (see Figure 1).

#### Variables coding

Prior to calculating effect sizes, the following information was extracted from the full texts of the studies: Author, publication year, sample size, manipulations of unitization (e.g., compound vs. noncompound words, related vs. unrelated pairs), stimulus types (e.g., word vs. picture stimuli), Language types (e.g., English alphabet vs. Chinese characters), age groups (e.g., younger vs. older adults), changes in the level of unitization (e.g., change vs. no-change), and key data for calculating effect sizes for associative memory, familiarity, and recollection estimates. When multiple independent experiments were reported in a single article, each experiment was coded in the analysis. All relevant information was coded twice by the same coder, with three-week intervals between coding, to measure test–retest reliability. Reliability statistics, using Kappa for categorical variables, exceeded 0.91 for all variables. Any discrepancies were resolved by revisiting the original literature.

#### Effect size calculation

Overall, the standard mean difference and 95% confidence intervals (CI) were computed as the effect size in the current study. However, because of different purpose and design, the original effect sizes extracted from each studies were various. Specifically, for study that manipulated the level of unitization as within-subject factor, the effect size can be computed directly when the *t* or *F* values (obtained in one-way, two level ANOVA, mixed analyses of variance) were reported (*Cohen’s d* = t/ $\sqrt{n}$ or = $\sqrt{\frac{F}{n}}$ ), whereas they were not reported, the effect size was calculated using the formula for repeated measures: *Cohen’s d_rm_* = $\frac{M_{diff}}{\sqrt{\frac{SD_{1}^{2}+SD_{2}^{2}-2rSD_{1}SD_{2}}{n}}}$ × $\sqrt{2\left(1-r\right)}$ , for which *M_diff_* is the mean difference between the high-and low-unitization conditions, *SD* is the standard deviation to each unitization conditions, and *r* is the correlation between the two unitization conditions. For study that manipulated the level of unitization as between-subject factor, the effect size was calculated using the formula for independent measures: *Cohen’s d_im_* = $\frac{M_{diff}}{\sqrt{\frac{\left(n_{1}-1\right)S_{1}^{2}+\left(n_{2}-1\right)S_{2}^{2}}{n_{1}+n_{2}-2}}}$ . When the data were not reported in these study, we contact the authors for assistance (*n* = 12). Due to the early publication, the data of one study had been lost (Rhodes and Donaldson, 2008).

#### Data analysis

Effect sizes were calculated for each study, as described in above section. The Comprehensive Meta-Analysis (CMA 3.0) program was used to combine effect sizes and calculate their 95% confidence intervals (CI). The *Q*-statistic was used to conduct significance tests on the basis of random-effects models and to estimate the heterogeneity of effect sizes across studies. *I*^2^ was computed to assess the amount of variation derived from heterogeneity. If the heterogeneity analysis was significant, subgroup analysis was performed to detect different effects of categorical moderators, using *Q*-tests with mixed-effect models. Subgroup comparisons were only conducted for moderator subgroups with at least four studies to ensure the analysis’ effectiveness. Additionally, we conducted exploratory analyses to assess whether the current data-set indicated any evidence of publication bias, with funnel plot and Fail-safe N (Rosenthal, 1979).

## Results

### Preliminary analysis

**Figure 2:**
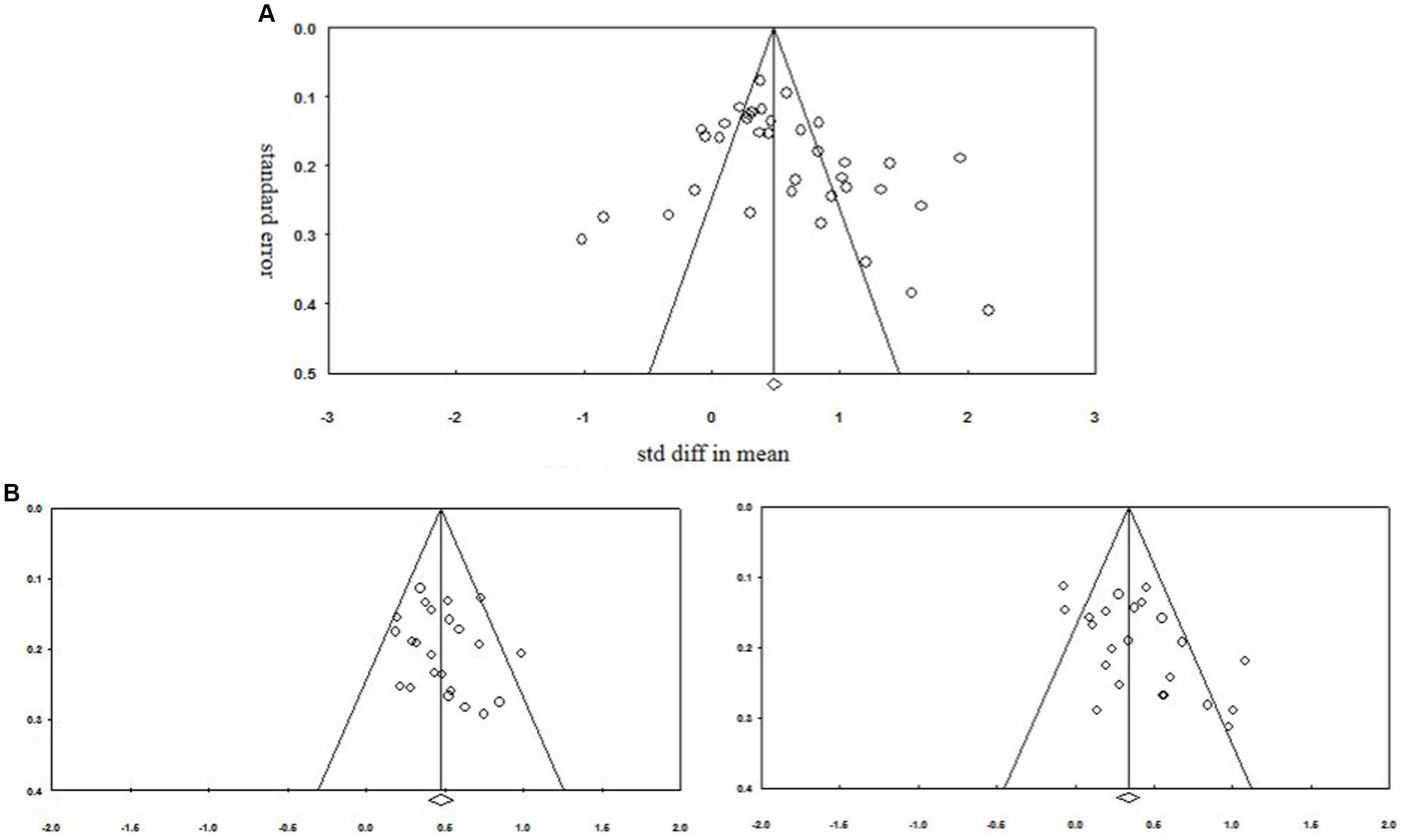
Funnel plots of effect sizes and standard errors of all studies included in the current analyses: **(A)** showing the associative memory; **(B)** showing the familiarity estimate; and **(C)** showing the recollection estimate.

In a funnel plot of all effect sizes (Figure 2), the effect sizes distributed relatively evenly around the combined weighted effect size, indicating an absence of a clear publication bias in the current data-set. Statistical asymmetry was, likewise, not indicated by the fail-safe N. Three thousand one hundred and five (for associative memory), 896 (for familiarity) and 611 (for recollection) additional studies with a *d* of 0, respectively, would reduce the magnitude of the overall effect sizes to non-significant (*p* = 0.05). These results indicate that the published results have evident value.

### Combined effect sizes

**Figure 3:**
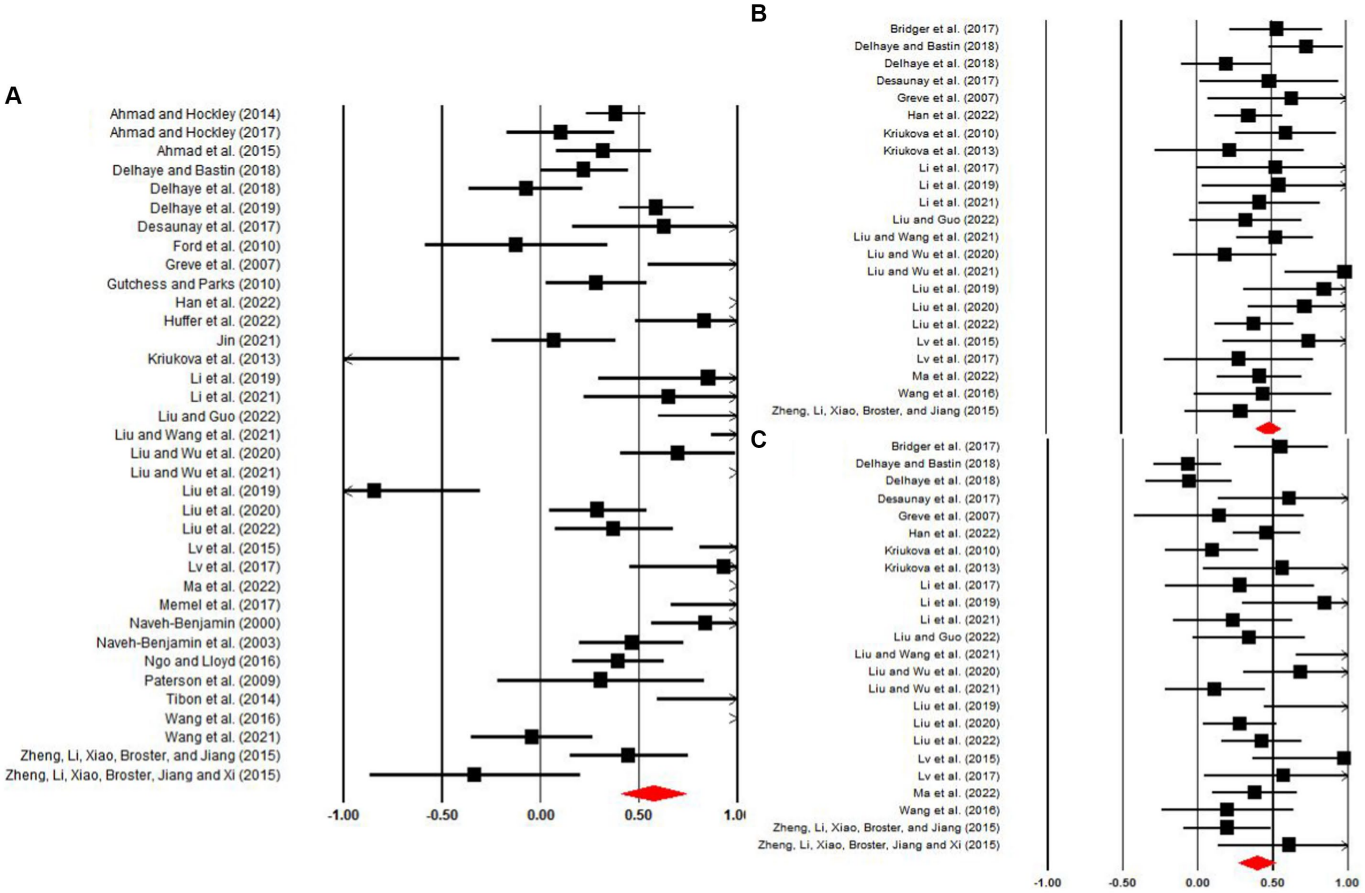
Effect sizes and forest plots for studies concerning the effects of unitization on **(A)** associative memory, **(B)** familiarity, and **(C)** recollection estimates. Red diamonds reflect the magnitude and 95% CI of the combined effect size.

Table 2 and Figure 3 show the meta-analysis results. The associative memory *d* was 0.57, indicating a significantly better associative memory under high unitization conditions compared to low unitization conditions (*p* < 0.001). Familiarity and recollection *d*s were 0.48 and 0.39, respectively, both statistically significant (*ps* < 0.001) and indicating larger estimates under high than low unitization conditions. These effect sizes met Cohen’s (1988) criteria for medium (0.5 < *d* < 0.8) and small (0.2 < *d* < 0.5) effects, respectively, providing clear support for the promotion effect of unitization on associative memory, familiarity, and recollection.

**Table 2:** Main parameters regarding the meta-analysis (random effects) of associative memory, familiarity and recollection estimates.

	Statistic test							
	Overall effect sizes					Heterogeneity		
Outcomes	*n*	Cohen’s *d*	95% CI	*Z*	*p*	*Q*_-Between_	*p*	*I^2^*
Associative memory	36	0.57	[0.41, 0.74]	6.92	< 0.001	300.21	< 0.001	88.34
Familiarity	23	0.48	[0.39, 0.59]	11.21	< 0.001	26.87	0.22	18.13
Recollection	24	0.39	[0.27, 0.51]	6.31	< 0.001	61.33	< 0.001	62.50

### Moderator analysis

The *Q-*statistics for associative memory and recollection *ds* were significant (associative memory: *Q* = 300.21, *p* < 0.0001, *I*^2^ = 88.34; recollection: *Q* = 61.33, *p* < 0.001, *I*^2^ = 62.50), suggesting that moderator analyses were necessary. In contrast, the non-significant *Q-*statistics for familiarity *d* (*Q* = 26.87, *p* = 0.22, *I*^2^ = 18.13) indicated that the positive effect of unitization on familiarity was stable and not influenced by other variables.

#### Associative memory

Table 3 presents the moderator analysis of associative memory. Firstly, manipulations of unitization significantly moderated the associative memory *d*, *Q*_-between_ statistic = 41.75, *p* < 0.001, with studies manipulating the level of unitization using compound vs. noncompound words showing significantly smaller *d* than studies using related vs. unrelated pairs. Secondly, stimulus types significantly moderated the associative memory *d*, *Q*_-between_ statistic = 27.54, *p* < 0.001, with studies using picture stimuli showing significantly larger *d* compared to studies using word stimuli. Thirdly, age groups did not moderate the associative memory *d*, *Q*_-between_ statistic = 0.75, *p* = 0.39, indicating that unitization can improve associative memory in both younger and older adults, with no significant differences between them. Fourthly, language types did not moderate the associative memory *d*, *Q*_-between_ statistic = 0.36, *p* = 0.55, indicating that unitization can improve associative memory for both English alphabets and Chinese characters, with no significant differences between them. Finally, changes in the level of unitization significantly moderated the associative memory *d*, *Q*_-between_ statistic = 4.19, *p* = 0.041, with studies that changed the level of unitization between studied and rearranged pairs showing significantly larger *d* than studies that did not change.

**Table 3:** The moderator analysis of associative memory *d*.

	Statistic test						
Moderators		*Q*_-within statistic_				*Q*_-between statistic_	
	*k*	Cohen’s *d*	95% CI	*Z*	*p*	*Q*_-Between_	*p*
Manipulations of unitization						41.75	<0.001
Compound vs. noncompound words	36	0.36	[0.18, 0.53]	4.04	< 0.001		
Related vs. unrelated pairs	27	0.83	[0.63,1.03]	8.05	< 0.001		
Stimulus types						27.54	<0.001
Word stimuli	47	0.43	[0.27, 0.58]	5.41	< 0.001		
Picture stimuli	16	0.95	[0.68, 1.21]	6.95	< 0.001		
Age groups						0.75	0.39
Older adults	13	0.55	[0.25, 0.85]	3.59	< 0.001		
Younger adults	50	0.56	[0.40, 0.72]	7.04	< 0.001		
Characters types						0.36	0.55
Chinese characters	21	0.51	[0.29, 0.72]	4.66	< 0.001		
English alphabets	26	0.36	[0.17, 0.55]	3.77	< 0.001		
Changes in the level of unitization						4.19	0.041
Change	24	0.66	[0.43, 0.89]	5.57	< 0.001		
No-change	38	0.51	[0.33, 0.69]	5.61	< 0.001		

*k*, number of study outcomes; Cohen’s *d*, effect size; 95% CI, 95% confidence interval around the point estimate of the effect size; *Z*, a statistic value to estimate the homogeneity within a set of studies; *Q*_-within_, a statistic testing for the homogeneity within a set of studies; Q_-between_, a moderation statistic testing for the significance of the contrast between different sets of studies.

#### Recollection estimate

Table 4 presents the moderator analysis of recollection estimate. Firstly, the results revealed that neither manipulations of unitization nor stimulus types moderated the recollection *d*, all *Q*_-between_ statistics <0.13, all *ps* > 0.72. This suggests that the impact of unitization on recollection estimate was consistent across all studies that manipulated the level of unitization with compound vs. noncompound words or related vs. unrelated pairs, and that used picture stimuli or word stimuli. Secondly, although age groups did not moderate the recollection *d*, *Q*_-between_ statistic = 0.77, *p* = 0.38, the *Q*_-within_ statistic revealed a different effects of unitization on recollection in younger and older adults, with a significant effect in younger adults (*Z* = 6.09, *p* < 0.001), but not in older adults (*Z* = 1.67, *p* = 0.09). Fourthly, language types significantly moderated the recollection *d*, *Q*_-between_ statistic = 14.87, *p* < 0.001, with a significant effect for Chinese characters (*Z* = 6.22, *p* < 0.001), but not for English alphabets (*Z* = 0.55, *p* = 0.58). Finally, changes in the level of unitization significantly moderated the recollection *d*, *Q*_-between_ statistic = 4.73, *p* = 0.030, with studies that changed the level of unitization showing significantly larger *d* than studies that did not change.

**Table 4:** The moderator analysis of recollection *d*.

	Statistic test						
Moderators	*Q*_-within statistic_					*Q*_-between statistic_	
	*k*	Cohen’s *d*	95% CI	*Z*	*p*	*Q*_-Between_	*p*
Manipulations of unitization						0.07	0.79
Compound vs. noncompound words	19	0.37	[0.22, 0.53]	4.71	< 0.001		
Related vs. unrelated pairs	17	0.34	[0.18, 0.51]	4.11	< 0.001		
Stimulus types						0.13	0.72
Word stimuli	25	0.35	[0.21, 0.49]	4.99	< 0.001		
Picture stimuli	11	0.38	[0.18, 0.58]	3.76	< 0.001		
Age groups						0.77	0.38
Older adults	5	0.25	[−0.04, 0.55]	1.67	0.09		
Younger adults	31	0.38	[0.26, 0.50]	6.09	< 0.001		
Characters types						14.87	< 0.001
Chinese characters	18	0.47	[0.32, 0.62]	6.22	< 0.001		
English alphabets	7	0.06	[−0.16, 0.28]	0.55	0.58		
Changes in the level of unitization						4.73	0.03
Change	17	0.47	[0.31, 0.64]	5.66	<0.001		
No-change	19	0.27	[0.12, 0.41]	3.53	<0.001		

## Discussion

This meta-analysis aimed to explore the effect of unitization on associative memory, along with potential moderators that may influence this effect. The results indicated that unitization can enhance overall memory performance and its supporting processes (i.e., familiarity and recollection). However, these promotion effects were subject to moderation by some factors, including the manipulations of unitization, stimulus types, language types, age groups and changes in the level of unitization. Specifically, the impact of unitization on associative memory is moderated by manipulations of unitization, stimulus materials, and changes in the level of unitization, with the latter having a significant influence on the role of unitization in recollection. Although the age groups did not influence the relationship between unitization and recollection, the *Q_-within_* statistic revealed that unitization augments recollection in younger adults, but this effect maybe absent in older adults. This implies that changes in the level of unitization and age groups are two important factors that may influence the impact of unitization on associative memory and recollection. However, due to the constraints of the meta-analysis methodology, we could not directly evaluate whether these two factors interact the unitization effects for associative memory and recollection. To address this question and better understand how these factors interact to affect the impact of unitization on associative memory, a mixed experimental design, with level of unitization and changes in the level of unitization as within-subject factors and age groups as a between-subject factor, was conducted.

## Behavioral study

### Methods

#### Transparency and openness

We provide a comprehensive account of the procedures utilized to determine the appropriate sample size. Additionally, we describe all of the manipulations, materials, and measures that were employed in the study. The pertinent information can be accessed at https://osf.io/sp9uv/files/osfstorage. The data underwent analysis using SPSS 25.0.

#### Participants

The necessary number of participants for this experiment was determined through power analyses conducted using G*Power software Version 3.1. Drawing from a related study (Liu et al., 2022), which reported an effect size of 0.66 for the interaction between the level of unitization and changes in the level of unitization, with α set at 0.05 and 1− β at 0.95, the minimum required sample size was determined to be 6 participants. A total of 70 participants were recruited for this experiment, including 35 undergraduate students (women = 23, mean age = 20.03 years, range: 18–22) and 35 community-dwelling older adults (women = 18, mean age = 64.26 years, range: 58–72). All participants were right-handed native Chinese speakers with normal or corrected-to-normal vision, and no reported history of memory impairment based on the delayed-recall component of the Montreal Cognitive Assessment test. The younger group had a mean of 13.26 years of education (range: 12–15), while the older group had a mean of 12.77 years of education (range: 9–15), with no significant difference between the two age groups [*t*_(68)_ = 1.40, *p* = 0.17]. All participants volunteered and received a compensation of ¥50 in total. Informed consent was obtained from all participants, and the protocol—Improving Associative Memory in Younger and Older Adults with Unitization: Evidence from Meta-analysis and Behavioral Studies—was approved by the Ethics Committee of the Institute of Department of Psychology at Shanghai Normal University.

#### Materials

Ninety-six compound words and 96 noncompound words were selected from a pool of stimuli utilized in previous studies (Liu et al., 2020a, 2022). Ten younger and ten older adults, who did not take part in the main experiment, were recruited to assess the level of familiarity and unitization of these word pairs, and results indicated that the experimental materials were well-matched in this study. The detailed information are shown in Supplementary Appendix 1.

#### Procedures

**Figure 4:**
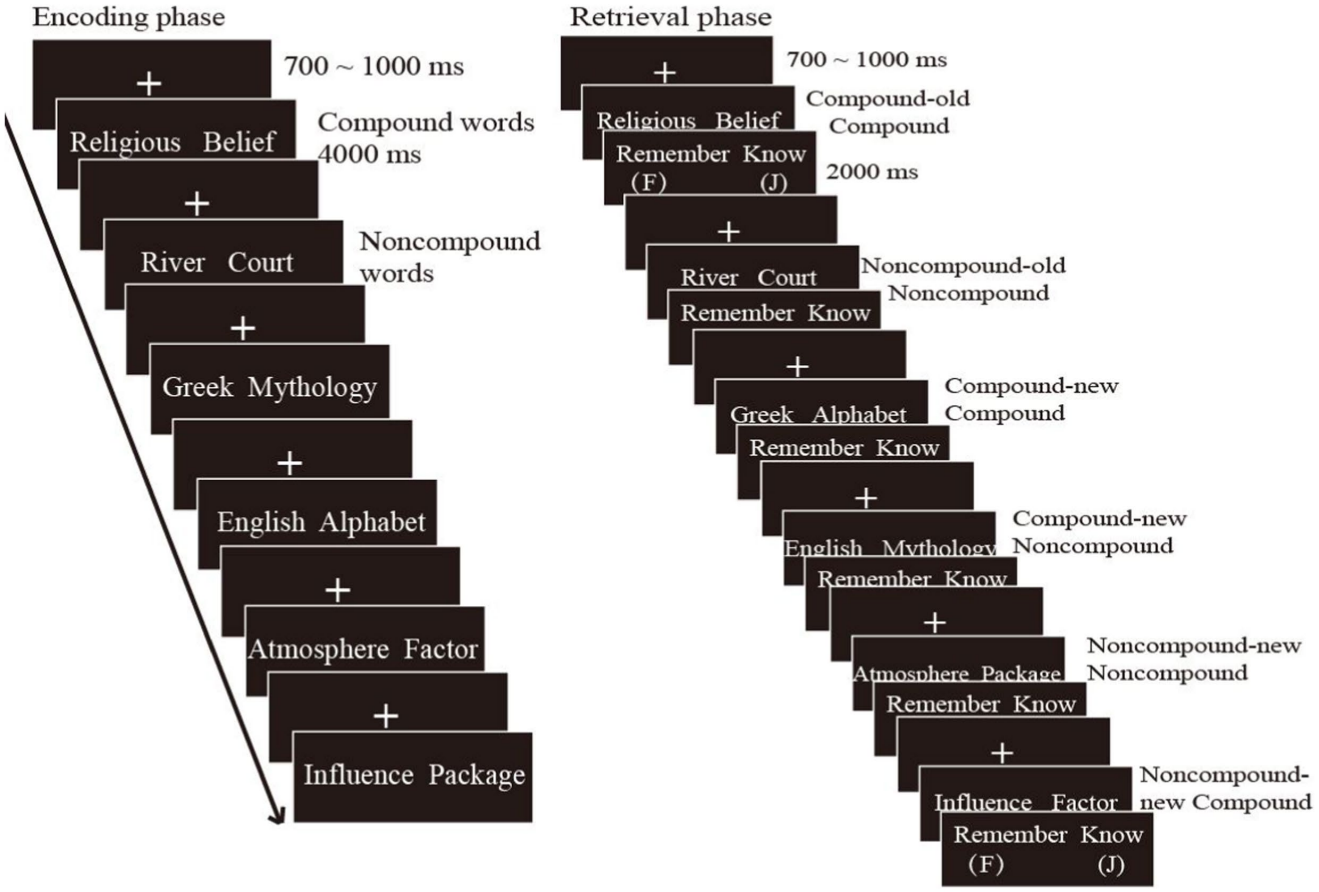
Illustration of the procedures with examples of studied word pairs at encoding (left) and tested word pairs at retrieval (right).

The whole experiment consisted of two blocks for younger adults, each of which contained an encoding and retrieval phases. During the encoding phase, 48 compound words and 48 noncompound words were presented randomly. Each trial began with a “+” presented on the central screen for 700–900 ms, followed by the word pairs presented for 4,000 ms. During this period, participants rated the level of unitization using a 5-point scale. After a two-minute break, a recognition test was conducted with 96 word pairs divided into six retrieval conditions: Compound-old compound (the word pairs that were CW at encoding were presented in the same configuration as during encoding, i.e., CW-old CW), compound-new compound (the word pairs that were CW at encoding were rearranged into new CW, i.e., CW-new CW), compound-new noncompound (the word pairs that were CW at encoding were rearranged into new NCW, i.e., CW-new NCW), noncompound-old noncompound (the word pairs that were NCW at encoding were presented in the same configuration as during encoding, i.e., NCW-old NCW), noncompound-new noncompound (the word pairs that were NCW at encoding were rearranged into new NCW, i.e., NCW-new NCW), and noncompound-new compound (the word pairs that were NCW at encoding were rearranged into new CW, i.e., NCW-new CW). Examples are illustrated in Supplementary Appendix 2. Each trial of the recognition test began with a “+” presented on the central screen for 700–900 ms, followed by the word pairs presented for 4,000 ms, during which the participants had to respond with either an “intact” or “rearranged” response. Then, to estimate the contribution of familiarity and recollection, the participants had to make a “remember” or “know” response within 2,000 ms. Participants were required to respond “remember” if they could recall specific details about the experience of studying the word pairs. In contrast, they were to respond “know” if they were unable to recall specific details about the word pairs but the pairs seemed familiar. If they were unable to complete the “intact” or “rearranged” judgment when the word pairs appeared, they could also supplement this response when the “remember/know” cue was subsequently presented. The procedure were exactly the same for older adults, except with 4 blocks. The procedures are shown in Figure 4.

#### Data pre-processing and analysis

**Figure 5:**
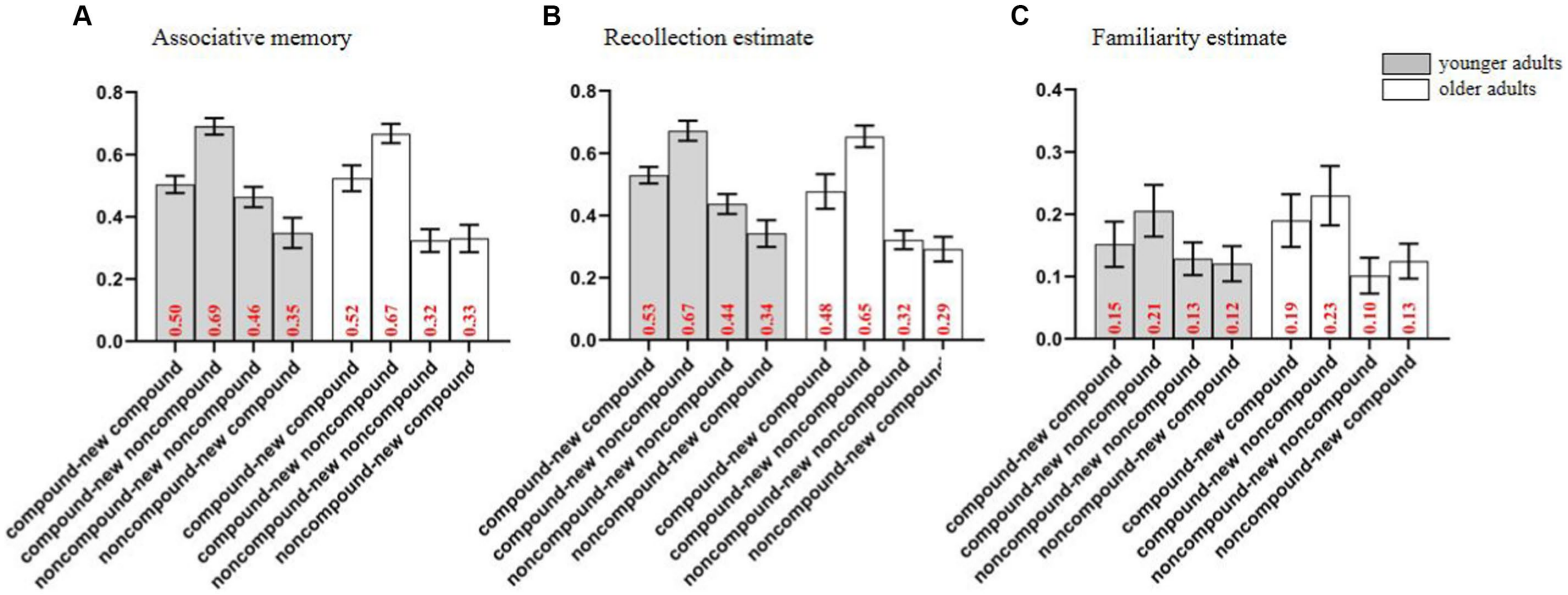
The associative memory **(A)**, recollection estimate **(B)**, and familiarity estimate **(C)** across different groups, levels and changes in unitization levels (M±SE). Error bars represent standard errors.

The unitization ratings at encoding, Hits and false alarms at retrieval are reported in Supplementary Appendix 3. In the main text, we focus exclusively on the impact of unitization, changes in the level of unitization, and age groups on associative memory and its supporting processes (i.e., familiarity and recollection estimates). Specifically, the overall performance of associative memory is equal to the Hits to intact pairs minus the false alarms to rearranged pairs. In addition, we collected RK judgments to assess the relative contribution of recollection and familiarity to associative memory. As suggested by Yonelinas (2002), we used an independent RK index (IRK) to compensate for the underestimation of actual familiarity in the proportion of “know” responses. In line with this method, the probability of giving a “remember” response was used as an index of recollection [Recollection = Hits(‘remember”)]. The index of familiarity, on the other hand, was calculated as the probability of an item receiving a “know” response, given that it was not recollected [Familiarity(IRK) = Hit(‘know”)/(1−Recollection)]. Consistent with the calculation of the overall performance of associative memory, the index of recollection estimate (i.e., the overall accuracy score for “remember” responses, see Figure 5B) were calculated by subtracting false alarms from Hits for “remember” responses, and the index of familiarity estimate which corrected by overall accuracy score for “know” responses were calculated by subtracting false alarms from corrected Hits (i.e., Familiarity(IRK)—false alarms for “know” response, see Figure 5C; Delhaye et al., 2018; Delhaye and Bastin, 2018; Liu et al., 2020b, 2021b). Furthermore, the rearranged pairs were utilized to establish the variable of changes in the level of unitization by determining whether there was a change or no-change in level of unitization between studied and rearranged pairs. While the old pairs did not contribute to this variable as it had not changed in itself. Specifically, when participants learned compound words and were tested on compound words (i.e., CW-new CW) or learn noncompound words and were tested on noncompound words (i.e., NCW-new NCW), the level of unitization between studied and rearranged pairs did not change, namely no-change conditions. Conversely, if participants learned compound words and were tested on non-compound words (i.e., CW-new NCW) or learned non-compound words and tested on compound words (i.e., NCW-new CW), the unitization level changed, namely change conditions.

For data analysis, we initially conduct three mixed ANOVAs, with level of unitization and changes in the level of unitization as the within-subject factors, and age groups as the between-subject factor, on overall performance of associative memory, familiarity and recollection estimates respectively, due to their lack of independence in variance. Secondly, to align with the results of the meta-analysis and to better understand the effect of unitization under different levels of moderators, we compare to performance of associative memory, familiarity and recollection estimates between compound and noncompound words under change and no-change conditions, and in older and younger adults separately, regardless of whether the three-way interactions are significant. Subsequently, we directly compare the unitization effect-equal to the performance under high unitization conditions minus that under low unitization conditions-between the change and no-change conditions for older and younger adults. Finally, in line with previous studies focusing on the relationship between unitization and group difference (i.e., age-related deficits), we also examine whether changes in the level of unitization modulates this relationship. That is, we investigated whether there are group differences for compound and noncompound words under change and no-change conditions.

Analysis were conducted using mixed analysis of variance (ANOVA) and follow-up tests were run using Bonferroni *post hoc* tests in the SPSS 25.0. Partial eta square was reported for ANOVA when the effects were significant. And once the *post hoc* analysis were not significant, Bayesian analysis were conducted to compare the null and alternate hypotheses, using JASP V0.14.0.0. The alpha level was set at 0.05.

## Results

### Overall performance of associative memory

The overall performance of associative memory (Hits—false-alarms, see Figure 5A) was submitted to a 2 × 2 × 2 mixed ANOVA with level of unitization (compound vs. noncompound) and changes in the level of unitization (no-change vs. change) as the within-subject factors, and age groups (younger vs. older) as the between-subject factor. The results showed significant main effects of level of unitization [*F*(1, 68) = 114.38, *p* < 0.001, $\;\eta_{p}^{2}$ = 0.63] and changes in the level of unitization [*F*(1, 68) = 16.47, *p* < 0.001, $\;\eta_{p}^{2}$ = 0.20], along with significant interactions between the level of unitization × changes in the level of unitization [*F*(1, 68) = 39.55, *p* < 0.001, $\;\eta_{p}^{2}$ = 0.37] and level of unitization × changes in the level of unitization × age groups [*F*(1,68) = 5.55, *p* = 0.021, $\;\eta_{p}^{2}$ = 0.08]. Subsidiary analyses were performed under the no-change and change conditions, or for younger and older adults, respectively.

Under the no-change conditions, a 2 × 2 mixed ANOVA with level of unitization as within-subject factors and age groups as between-subject factor revealed significant main effect of level of unitization [*F*(1, 68) = 25.88, *p* < 0.001, $\;\eta_{p}^{2}$ = 0.28] and interaction [*F*(1, 68) = 11.28, *p* < 0.001, $\;\eta_{p}^{2}$ = 0.14]. Further analysis revealed that younger adults had equivalent associative memory between the compound and noncompound words (*p* = 0.23, *BF*_10_ = 0.25), while older adults performed better for compound words compared to noncompound words (*p* < 0.001). Similarly, a 2 × 2 mixed ANOVA was conducted under the change conditions. The only significant effect was a main effect of level of unitization [*F*(1, 68) = 119.03, *p* < 0.001, $\;\eta_{p}^{2}$ = 0.64]. Both younger and older adults performed better for compound words than for noncompound words. The main effect of age groups [*F*(1, 68) = 0.22, *p* = 0.64] and interaction [*F*(1, 68) = 0.006, *p* = 0.94] failed to reach significance.

For younger adults, a 2 × 2 repeated-measures ANOVA, with factors of level of unitization and changes in the level of unitization, was conducted. The results revealed significant main effect of level of unitization [*F*(1, 34) = 43.31, *p* < 0.001, $\;\eta_{p}^{2}$ = 0.56] and interaction [*F*(1,34) = 31.01, *p* < 0.001, $\;\eta_{p}^{2}$ = 0.48]. Further analysis revealed that the unitization effect (i.e., the difference between the high and low unitization conditions) was greater under the change conditions compared to the no-change conditions [*t*(34) = 5.57, *p* < 0.001, *d* = 0.94]. The same is true for the analysis of older adults, a 2 × 2 repeated-measures ANOVA revealed main effects of level of unitization [*F*(1, 34) = 71.84, *p* < 0.001, $\;\eta_{p}^{2}$ = 0.68] and changes in the level of unitization [*F*(1, 34) = 18.71, *p* < 0.001, $\;\eta_{p}^{2}$ = 0.36], along with a significant interaction [*F*(1, 34) = 9.73, *p =* 0.004, $\;\eta_{p}^{2}$ = 0.22]. Further analysis likewise revealed a greater unitization effect under the change conditions [*t*(34) = 3.12, *p* = 0.004, *d* = 0.53].

Regarding the group difference, it only appeared for noncompound words under the no-change conditions (i.e., participants learned noncompound words and were tested on noncompound word, NCW-new NCW), with younger adults exhibiting a higher score compared to older adults [*t*(34) = 2.63, *p* = 0.011, *d* = 0.44]. While under the other three conditions (i.e., CW-new CW, CW-new NCW, and NCW-new CW), no significant group difference was observed [all *t*s(34) < 0.58, *ps > 0*.59, *BF_10_ >* 0.28].

### Recollection and familiarity estimates

For recollection estimate (see Figure 5B), a 2 × 2 × 2 mixed ANOVA revealed significant main effects of level of unitization [*F*(1, 68) = 87.13, *p* < 0.001, $\;\eta_{p}^{2}$ = 0.56] and changes in the level of unitization [*F*(1, 68) = 8.88, *p* = 0.004, $\;\eta_{p}^{2}$ = 0.12], along with a significant interaction between these two factors [*F*(1, 68) = 55.08, *p* < 0.001, $\;\eta_{p}^{2}$ = 0.45]. Further analysis showed that participants exhibited larger recollection estimate for compound words than for noncompound words under the both change and no-change conditions (all *ps* < 0.001), with a larger unitization effect under the change conditions compared to the no-change conditions (*p* < 0.001). To more accurately assess the effects of these two factors on recollection estimate in different groups, separate analysis were conducted for younger and older adults. The results showed that both younger and older adults exhibited larger recollection estimate for compound words than for noncompound words, regardless of whether the conditions involved a change or no-change (all *ps* < 0.039). Similarly, the unitization effects were larger under the change conditions compared to the no-change conditions for both younger [*t*(34) = 5.60, *p* < 0.001, *d* = 0.95] and older adults [*t*(34) = 4.90, *p* < 0.001, *d* = 0.83]. Regarding the group difference, the results are consistent with the above findings on associative memory. Older adults only exhibited smaller recollection estimate for noncompound words under the no-change conditions [*t*(34) = 2.63, *p* = 0.011, *d* = 0.44]. While under the other three conditions (i.e., CW-new CW, CW-new NCW, and NCW-new CW), no significant group difference was observed [all *t*s(34) < 0.86, *ps > 0*.39, *BF_10_ >* 0.33].

For familiarity estimate, a 2 × 2 × 2 mixed ANOVA revealed significant main effects of level of unitization [*F*(1, 66) = 7.51, *p* = 0.008, $\;\eta_{p}^{2}$ = 0.10] and changes in the level of unitization [*F*(1, 66) = 11.68, *p* = 0.001, $\;\eta_{p}^{2}$ = 0.15], along with an interaction between the level of unitization and changes in the level of unitization [*F*(1, 66) = 9.47, *p* = 0.003, $\;\eta_{p}^{2}$ = 0.13]. Further analysis revealed participants exhibited larger familiarity estimate for compound words than for noncompound words under the both change and no-change conditions (all *ps* < 0.045, see Figure 5C), with a larger unitization effect under the change conditions compared to the no-change conditions (*p* = 0.003). Similarity, separate analyses were conducted for younger and older adults. The results showed that younger adults exhibited larger familiarity estimate for compound words than for noncompound words under the change conditions only (*p* = 0.046), while older adults exhibited larger familiarity estimate for compound words under the both change and no-change conditions (*ps* < 0.046). Additionally, further analysis revealed that younger adults exhibited a larger unitization effect under the change conditions [*t*(34) = −3.55, *p* = 0.001, *d* = −0.60], while the unitization effects between the change and no-change conditions were equivalent for older adults [*t*(32) = −0.96, *p* = 0.35, *BF*_10_ = 0.28]. No significant group difference was found among the four conditions [all *t*s(34) < 0.70, *ps > 0*.49, *BF_10_ >* 0.30].

## Discussion

To solve the problems left in the meta-analysis: How age groups and changes in the level of unitization interact to affect the impact of unitization on associative memory and its processes, a behavioral experiment, with level of unitization and changes in the level of unitization as the within-subject factors and age groups as a between-subject factor, was conducted. The significant interactions between the level of unitization, changes in the level of unitization, and age groups demonstrated the complex interplay between these factors in influencing associative memory. Specifically, unitization could enhance younger and older adults’ associative memory and its supporting processes (i.e., familiarity and recollection) when the level of unitization between studied and rearranged pairs changed. However, when this level remained constant, unitization exhibited no impact on associative memory and familiarity in younger adults, but showed an enhanced effect in older adults. Furthermore, results revealed a marked group difference when participants learned noncompound words and were tested on noncompound words. Compared to younger adults, older adults showed lower scores in associative memory and recollection estimate. Once breaking this condition and involving compound words, older adults performed an equivalent associative memory compared to younger adults, and the group difference in familiarity and recollection estimates also disappeared. These findings seem to imply that older adults can still remember well the associative knowledge they have already acquired. However, when it comes to newly association, older adults show significantly lower performance, primarily due to impaired recollection.

## General discussion

The impact of unitization on associative memory and recollection estimate remain a topic of debate, despite widespread agreement that familiarity can support associative memory when the to-be-learned items are “unitized” into a new representation. This study aimed to address these inconsistencies by exploring the potential moderators and examining how unitization affects associative memory and its processes under different conditions. Initially, a meta-analysis was conducted to identify the potential moderator. Results showed that changes in the level of unitization and age groups are two important moderators. Subsequently, a behavioral experiment was conducted to further examine how these two factors interact to influence the effect of unitization on associative memory. Overall, results suggested that unitization could enhance associative memory by increasing familiarity and recollection in both younger and older adults. However, the degree of these beneficial effects was influenced by changes in the level of unitization. Relative to the no-change conditions, in which the level of unitization between studied and rearranged pairs remained constant, the enhancement of associative memory and its processes via unitization was more larger under the change conditions for both age groups. In the following sections, we will elaborate on these findings separately.

### The effects of level of unitization and changes in the level of unitization in younger and older adults

In our study, we initially focused on the effects of level of unitization on associative memory and its processes, as this has been the focus of most previous studies. Our meta-analysis and behavioral studies consistently demonstrated that unitization can improve associative memory by increasing familiarity and recollection (see the significant *Q_-statistic_* in meta-analysis and main effect of level of unitization in behavioral study). However, previous empirical studies have often overlooked an important factor: Changes in the level of unitization between studied and rearranged pairs. Some studies matched this level (i.e., no-change conditions), while others did not (i.e., change conditions, see the column of “changes in the level of unitization” in Table 1). In this study, our second exploration focused on understanding how changes in the level of unitization influence the relationship between unitization and associative memory, utilizing a combined approach of meta-analysis and behavioral experimentation. The significant moderating analyses in meta-analysis and the observed interactions between the level of unitization and changes in the level of unitization in behavioral experiment substantiated our hypotheses. More specifically, for younger adults, unitization may or may not influence associative memory under the no-change conditions,^3^ while it always enhanced associative memory under the change conditions. In contrast, for older adults, unitization improved associative memory under the both change and no-change conditions. Moreover, inter-condition comparisons revealed that both younger and older adults exhibited more substantial unitization effects for associative memory under the change conditions compared to the no-change conditions. These findings were in line with the results from our meta-analysis or preexisting literature (Delhaye et al., 2019; Liu et al., 2020a,b, 2022). When distinguishing the contributions of familiarity and recollection, our meta-analysis and behavioral experiment consistently revealed a significant moderating effect of changes in the level of unitization. Specifically, regardless of the change or no-change conditions, unitization was found to enhance recollection estimate, thereby supporting associative memory in both age groups, with a greater unitization effect under the change conditions. As for the familiarity estimate, younger adults exhibited larger familiarity estimate only under the change conditions, while older adults demonstrated larger familiarity estimate under the both conditions. Inter-condition comparisons revealed that older adults demonstrated a comparable unitization effect between the two conditions, whereas younger adults exhibited a greater unitization effect under the change conditions. These findings about inter-condition comparisons align with the results from meta-analysis concentrating on the unitization effect on familiarity. In comparison to the no-change conditions, the effect size for familiarity was greater under the change conditions (change: *k* = 16, *d* = 0.57, 95% *CI* = [0.43, 0.72]; no-change: *k* = 19, *d* = 0.43, 95% *CI* = [0.31, 0.56], *Q_-between_* = 3.92, *p* = 0.048). Subsequently, we further explained these results from the perspectives of familiarity and recollection, respectively.

### The contribution of familiarity to associative memory

Bader et al. (2010) proposed that familiarity can be categorized into two types: Pre-experimental and experimental. Pre-experimental familiarity is an absolute or baseline signal that depends on the materials (e.g., the terms “Greek Alphabet” and “Influence Factor,” which were not previously studied during encoding but presented together in daily life), while experimental familiarity benefits from learning (e.g., the terms “River Count, which were previously studied during encoding but not presented together in daily life) (Mackenzie and Donaldson, 2007; Stenberg et al., 2009; Bader et al., 2010; Delhaye et al., 2019). Compound words, which are formed through repeated daily experiences, consistently evoke higher pre-experimental familiarity compared to noncompound words. In this study, the compound and noncompound words learned during encoding were divided into six retrieval conditions, as shown in Figure 4 and Supplementary Appendices 2, 4. The compound-intact pairs acquired both pre-experimental and experimental familiarity, as they were presented together frequently in daily life and learned at encoding. The noncompound-intact pairs had experimental familiarity only, as they were learned at encoding. The compound-new noncompound and noncompound-new noncompound words had no pre-experimental or experimental familiarity since they were neither presented together in daily life nor learned at encoding. In contrast, the compound-new compound and noncompound-new compound words had pre-experimental familiarity only since they were presented together in daily life but not learned during encoding (see Supplementary Appendix 4). During the task of distinguishing between intact and rearranged pairs, the pre-experimental or experimental familiarity worked. For instance, when participants were asked to differentiate between the compound-intact and compound-new compound words, or between the noncompound-intact and noncompound-new noncompound words, experimental familiarity was the key factor influencing their ability to distinguish between these two pairs, after excluding the influence of similar pre-experimental familiarity. On the other hand, when participants were required to distinguish between the compound-intact and compound-new noncompound words, or between the noncompound-intact and noncompound-new compound words, both pre-experimental and experimental familiarity contributed to their ability to differentiate between these two pairs. Therefore, there was a greater familiarity estimate under the change conditions than under the no-change conditions.

### The contribution of recollection to associative memory

Repeated experiences in daily life create a stronger association between the sub-members of compound compared to noncompound words, which help participants recall the original learned pairs from individual words (Liu et al., 2020a, 2022). For example, participants can recall “Greek-mythology” or “Greek-alphabet” based on “Greek.” This process involves recollection-reject or recollection-accept processing (Dobbins et al., 1998; Rotello et al., 2000; Migo et al., 2009). As shown in Figure 4, when participants were required to distinguish between the compound-intact and compound-new compound words, or between the noncompound-intact and noncompound-new noncompound words, the stronger association within compound words allowed participants to recall the learned words based on the individual words and then accept the intact pairs or reject the recombined pairs (see Supplementary Appendix 3). Therefore, compound words always demonstrated higher recollection estimate than noncompound words. Similarly, when participants were asked to distinguish between the compound-intact and compound-new noncompound words, or between the noncompound-intact and noncompound-new compound words, compound words still recalled the original pairs based on the stronger association, resulting in greater recollection estimate than noncompound words. It is important to note that the compound-new compound words had stronger associations and higher pre-experimental familiarity than the compound-new noncompound words. This may have made participants more likely to falsely accept the compound-new compound words, leading to a smaller unitization effect on recollection under this condition. Overall, the enhanced contributions of familiarity and recollection under the change versus no-change conditions promote the overall performance of associative memory.

### The effects of level of unitization and changes in the level of unitization on group difference

In assessing the influence of age on associative memory, we unexpectedly found that the performance of associative memory between older and younger adults varied depending on the interaction between the level of unitization and changes in the unitization level. Compared to other conditions (e.g., compound-new compound, compound-new noncompound, noncompound-new compound words), the only condition (i.e., noncompound-new noncompound words) revealed significant group difference, suggesting potential impairment of associative memory in older adults. Drawing on prior research (Ahmad et al., 2015; Memel and Ryan, 2017; Delhaye et al., 2018, 2019; Delhaye and Bastin, 2018; Huffer et al., 2022), our initial comparison focused on the effect of unitization on group difference under the no-change conditions. Results indicated that older adults exhibited equivalent associative memory to younger adults for compound words, as opposed to their lower associative memory for noncompound words. This reduction of group difference was characterized by a higher proportion of Hits to compound words in older adults (see Supplementary Appendix 3), likely attributable to their increased pre-experimental and experimental familiarity, along with the stronger associations of the compound-intact pairs. When evaluating the contribution of familiarity and recollection, although no main and interaction effects involving age groups were found, planned comparison revealed that older adults, compared to younger adults, exhibited smaller recollection estimate for noncompound words but not for compound words. Furthermore, no significant group difference in familiarity estimate was observed for either compound or noncompound words. These result were consistent with the study of Delhaye and Bastin (2018) and supported the view that older adults showed impaired recollection but relatively intact familiarity. Under the change conditions, the performance of older adults was comparable to that of their younger counterparts. This reduction of group difference was characterized not only by higher Hits to compound-intact pairs but also by fewer false alarms to compound-new noncompound words in older adults (see Supplementary Appendix 3). In differentiating between the contributions of familiarity and recollection, no significant group difference was noted for either compound or noncompound words. This could be explained by the ability of older adults to accurately recall original pairs post-learning compound words, regardless of whether these pairs were presented as intact or rearranged pairs, similar to the abilities of the younger adults. In summation, older adults exhibited difficulty in accurately recollecting pairings when tasked with learning noncompound, evidenced by a reduced recollection estimate. The presence of a preexisting association, however, aids their recollection to some extent when learning compound words. In terms of familiarity processes, no significant variation was observed between age groups for both compound and noncompound words in either conditions, which supports the viewpoint that familiarity in older adults remains relatively intact. Additionally, the current study demonstrates that both younger and older adults exhibit optimal associative memory when learning compound words and testing non-compound words. In this scenario, the increased pre-experimental and experimental familiarity and stronger association within compound-intact pairs, as well as the lower pre-experimental familiarity within compound-new noncompound words, all help participants differentiate between them.

### Limitations

Although this study is the first to combine meta-analysis and behavioral experiments to investigate the effects of unitization and changes in the level of unitization on associative memory in older and younger adults, it also has its limitations. Firstly, the meta-analysis includes only studies that manipulated the level of unitization by using compound words/related pairs vs. unrelated pairs, while excluding studies that manipulated the level of unitization from other strategies, such as concept definition vs. sentence frame tasks or interactive imagery vs. Item imagery tasks. The former is called bottom-up unitization, while the latter is known as top-down unitization (Tibon et al., 2014; Liu et al., 2022). Compared to bottom-up unitization, top-down unitization is more akin to how we naturally learn and form new associations in our daily lives. However, considering that (1) only one study investigated the impact of top-down unitization on associative memory in older adults (i.e., concept definition vs. Sentence frame tasks, Kamp et al., 2018), and (2) top-down unitization cannot determine changes in the level of unitization, we ultimately exclude these studies from the meta-analysis. Regarding the second point, specifically, during the encoding phase, participants are required to learned the random word pairs either as compound words (high unitization condition) or as separate words (low unitization condition), and then at retrieval, these random word pairs are rearranged into new random pairs (i.e., low unitization condition). In that case, the level of unitization between learned and rearranged pairs changes in high unitization condition. Conversely, the level of unitization does not change in low unitization condition. We cannot categorize this manipulations of unitization as a change or no-change conditions. Therefore, we have ultimately decided to exclude these studies from the mate-analysis. Future research could further include top-down unitization studies to examine the effect of unitization on associative memory and its processes. Secondly, compared to existing research on the impact of unitization on associative memory in older adults (mean > 70 years), the older participants recruited in this study are relatively young, with an average age of less than 65 years (mean = 64.26 years). This may have a certain impact on the experimental results, especially in recollection estimate. This is because cognitive function tends to show a noticeable decline with age, particularly in individuals over the age of 65. The lack of group differences on compound words and recollection estimate in this study may be due to this reason. Future research could recruit older participants with an older age range, or recruit older participants with mild cognitive impairment to further investigate the impact of unitization on their associative memory. Thirdly, the meta-analysis revealed that stimulus types significantly moderated the associative memory *d,* with studies using picture stimuli showing larger *d* compared to studies using word stimuli. However, there was no significant moderating effect observed for recollection *d*, which is somewhat beyond our expectations. This may be due to two differences between the stimulus: (1) Compared to word stimuli which only involve semantic encoding, picture stimuli involve both semantic and image encoding, with image encoding potentially enhancing associative memory. (2) The unitization levels of word and picture stimuli may be different. Studies using picture stimuli manipulates unitization levels by presenting objects in spatial configurations that are either plausible or implausible (Gutchess and Park, 2009; Tibon et al., 2014; Bridger et al., 2017; Delhaye et al., 2018; Huffer et al., 2022), or by showing objects that are either functionally related or unrelated (Ma et al., 2021; Liu et al., 2021a,b; Han et al., 2022). On the other hand, research using word stimuli manipulates the level of unitization by presenting compound words/related pairs versus unrelated words pairs. In particular, for compound words, the co-occurrence of their sub-members and the strong association between them enable participants to recall the compounds they initially learned based on the individual words. This process enhances the recollection estimate and counteracts the advantage of image encoding. Future research could further match the level of unitization between word and picture stimuli to investigate the moderating effect of stimulus types on the unitization effect. Finally, the language types significantly moderate the unitization effect on recollection *d*, with a facilitating effect on Chinese characters but no effect on English alphabets. After reviewing these literatures, it appears that most studies using English alphabets (*n* = 5/7) controlled for the changes in the level of unitization, while most studies using Chinese characters (*n* = 13/18) did not control for this level.

## Conclusion

In conclusion, this study found that the effect of unitization on associative memory was moderated by changes in the level of unitization and age groups, as indicated by a meta-analysis and a behavioral study. Among younger adults, unitization may or may not enhance associative memory under the no-change conditions, but significantly improved memory performance under the change conditions by increasing the contributions of familiarity and recollection. In contrast, older adults experienced an overall improvement in associative memory through unitization, which bolstered the contributions of familiarity and recollection in both conditions. When comparing the unitization effect between the change and no-change conditions, both younger and older adults showed a larger unitization effect under the change conditions. Furthermore, behavioral experiment revealed a marked group difference in associative memory when the unitization level of noncompound words remained unaltered. Upon breaking this condition, the group difference was reduced by enhancing familiarity or recollection. These findings not only clarify some of the inconsistencies in the literature concerning the impact of unitization on associative memory, but also suggest that unitization is a beneficial strategy for reducing group difference in associative memory, with its effectiveness varying according to the level of unitization changes.

## Data availability statement

The datasets presented in this study can be found in online repositories. The names of the repository/repositories and accession number(s) can be found at: the data and materials can be available from https://osf.io/sp9uv/files/osfstorage.

## Ethics statement

Informed consent was obtained from all participants, and the protocol—Improving Associative Memory in Younger and Older Adults with Unitization: Evidence from Meta-analysis and Behavioral Studies—was approved by the Ethics Committee of the Institute of Department of Psychology at Shanghai Normal University. The studies were conducted in accordance with the local legislation and institutional requirements. Written informed consent for participation in this study was provided by the participants’ legal guardians/next of kin.

## Author contributions

ZL: Conceptualization, Data curation, Formal analysis, Funding acquisition, Investigation, Methodology, Project administration, Resources, Software, Supervision, Validation, Visualization, Writing – original draft, Writing – review & editing. YW: Data curation, Writing – review & editing. YZ: Data curation, Writing – review & editing. JY: Conceptualization, Investigation, Writing – review & editing. WL: Conceptualization, Formal analysis, Supervision, Validation, Visualization, Writing – review & editing.

## Footnotes

^1^Many ERP studies have provided electrophysiological evidence for familiarity and recollection processes, with two distinct ERP components. Specifically, the bilateral frontal old/new effect (FN400 effect), which has an early onset (300–500 ms) and is maximal over bilateral frontal electrodes, has been associated with familiarity. In contrast, a later-onset old/new effect (500–800 ms; LPC effect) maximal over left parietal electrodes has been associated with recollection (Curran, 2000; Curran and Cleary, 2003; Rugg and Curran, 2007).

^2^The reasons for doing this are explained in the “limitation” section.

^3^Unitization could improve associative memory in meta-analysis (*n* = 20, *d* = 0.50, 95% *CI* = [0.29, 0.70]), but had no effect on empirical studies (Ahmad et al., 2015; Delhaye et al., 2019; Liu et al., 2020a,b, 2022; and this behavioral experiment).
