# Supplementary material for: Improving associative memory in younger and older adults with unitization: evidence from meta-analysis and behavioral studies
Source: Front Aging Neurosci. 2024 May 23;16:1389957. doi: 10.3389/fnagi.2024.1389957 (PMC11153858; doi:10.3389/fnagi.2024.1389957)
Supplement: Supplementary file 1 [file Data_Sheet_1.docx]

Appendices A

To assess the level of familiarity and unitization of these word pairs, ten younger and ten older adults were recruited. For familiarity rating, each individual word was rated on a scale of 1 to 5 (1 for very unfamiliar, 3 for moderately familiar, and 5 for very familiar), and the means of the two words in each pair were computed to determine the level of familiarity for the word pair. For unitization rating, the word pairs were presented together, and participants were asked to rate each pair on a scale of 1 to 5 (1 for low level of unitization, 3 for moderate level of unitization, and 5 for high level of unitization). The rating instructions were to rate the extent to which a pair of words can be coded as a whole. Higher level of unitization indicates a stronger relationship between the two words, similar to an idiom. Conversely, the lower the level of unitization, the weaker the relationship between the two words. At retrieval, the compound and noncompound words learned during encoding were recombined into new word pairs, which could be compound or noncompound words. These recombined pairs were also rated.

The following were our results: (1) At encoding, a 2 × 2 mixed ANOVA was conducted on the level of familiarity, with age groups (younger, older) as the between-subject factor and level of unitization (compound, noncompound) as the within-subject factor, which showed no significant effects (all *Fs* < 1.99; all *ps* > 0.18). However, for the level of unitization, a 2 × 2 mixed ANOVA revealed a significant main effect of level of unitization only [*F*(1, 18) = 941.17, *p* < 0.001,$\boldsymbol{\eta}_{\boldsymbol{p}}^{\boldsymbol{2}}$ = 0.98], with higher level of unitization observed for compound words than for noncompound words. (2) At retrieval, a 2 × 2 × 3 mixed ANOVA was conducted on the level of familiarity, with age groups (younger, older) as the between-subject factor and level of unitization (compound, noncompound), retrieval condition (intact, recombined to new compound, recombined to new noncompound) as the within-subject factors, which showed no significant effects (all *Fs* < 2.17; all *ps* > 0.13). Regarding the level of unitization, the results showed significant main effects of level of unitization [*F*(1, 18) = 366.47, *p* < 0.001,$\boldsymbol{\eta}_{\boldsymbol{p}}^{\boldsymbol{2}}$ = 0.95] and retrieval condition [*F*(1, 18) = 28.72, *p* < 0.001,$\boldsymbol{\eta}_{\boldsymbol{p}}^{\boldsymbol{2}}$ = 0.62] , along with a significant interaction between them [*F*(2, 36) = 948.43, *p* < 0.001,$\boldsymbol{\eta}_{\boldsymbol{p}}^{\boldsymbol{2}}$ = 0.99] . All other effects were not significant (all *Fs* < 1.68; all *ps* > 0.20). Follow-up analysis revealed that compound-intact and compound-new compound words induced higher level of unitization than the compound-new noncompound words. Conversely, the noncompound-intact and noncompound-new noncompound words induced lower level of unitization than the noncompound-new compound words. Additionally, the level of unitization between the compound-intact and compound-new compound (*p* = 0.30) and between the noncompound-intact and noncompound-new noncompound (*p* = 0.18) were not significant. These results indicate that the experimental materials were well-matched in this study.

Appendices B

Examples of the materials for CW and NCW at encoding and retrieval

| Encoding phase | | Retrieval phase | |  |
| --- | --- | --- | --- | --- |
| Encoding pairs | Examples at study | Examples at test | Retrieval pairs | Changes in the level of unitization |
| CW | ‘Religious Belief’  ‘宗教 信仰’ | ‘Religious Belief’  ‘宗教 信仰’ | CW-intact | -- |
|  | ‘Greek Mythology’  ‘希腊 神话’ | ‘Greek Alphabet’  ‘希腊 字母’ | CW-new CW | No-change |
|  | ‘English Alphabet’  ‘英语 字母’ | ‘English Mythology’  ‘英语 神话’ | CW-new NCW | Change |
| NCW | ’ River Court’  ‘河流 法庭’ | ’ River Court’  ‘河流 法庭’ | CW-intact | -- |
|  | ‘Atmosphere Factor’  ’气氛 因素’ | ‘Atmosphere Package’  ‘气氛 包裹’ | NCW-new NCW | No-change |
|  | ‘Influence Package’  ‘影响 包裹’ | ‘Influence Factor’  ’影响 因素’ | NCW-new CW | Change |

Note: CW means compound words, NCW means noncompound words. The rearranged pairs are utilized to establish the variable of changes in the level of unitization by determining whether there is a change or no change in level of unitization between the studied and rearranged pairs.

Appendices C

Unitization ratings at encoding: About unitization ratings during the encoding phase, a 2 (level of unitization: compound, noncompound) × 2 (age groups: younger, older) mixed ANOVA revealed only a significant main effect of level of unitization [compound: 4.79±0.04; noncompound words: 1.62±0.04, *F*(1,68) = 5077.11, *p* < .001, $\boldsymbol{\eta}_{\boldsymbol{p}}^{\boldsymbol{2}}$ = .99], while the main effect of age groups [*F*(1,68) = 1.10, *p* = .30] and interaction [*F*(1,68) = 0.87, *p* = .35] failed to reach significance.

Hits and false alarms: The Hits and false alarms across groups and conditions are shown in Table C1. First, we conducted a 2 × 2 mixed ANOVA on the Hits to intact pairs, with level of unitization (compound, noncompound) as the within-subject factor and age groups (younger, older) as the between-subject factor. It revealed a main effect of level of unitization only [*F*(1, 68) = 93.48, *p* < 0.001,$\boldsymbol{\eta}_{\boldsymbol{p}}^{\boldsymbol{2}}$= 0.59]. Follow-up analysis showed higher Hits to compound-intact pairs than to noncompound-intact pairs in both younger and older adults (all *ps* < 0.001). And then, we conducted a 2 × 2 × 2 mixed ANOVA on the false alarms to rearranged pairs, with level of unitization and changes in the level of unitization (change, no-change) as the within-subject factors and age groups as the between-subject factor. It revealed significant main effects of level of unitization [*F*(1, 68) = 11.71, *p <* 0.001,$\boldsymbol{\eta}_{\boldsymbol{p}}^{\boldsymbol{2}}$ = 0.15] and changes in the level of unitization [*F*(1, 68) = 16.47, *p <* 0.001,$\boldsymbol{\eta}_{\boldsymbol{p}}^{\boldsymbol{2}}$ = 0.20], along with interactions between level of unitization × changes in the level of unitization [*F*(1, 68) = 39.55, *p <* 0.001,$\boldsymbol{\eta}_{\boldsymbol{p}}^{\boldsymbol{2}}$ = 0.37] and level of unitization × changes in the level of unitization × age groups [*F*(1, 68) = 5.55, *p =* 0.021,$\boldsymbol{\eta}_{\boldsymbol{p}}^{\boldsymbol{2}}$ = 0.08]. Decomposition of the interaction revealed that younger adults reported higher false alarms to compound-new compound words than to noncompound-new noncompound words (*p* < 0.001), but they reported lower false to compound-new noncompound words than to noncompound-new compound words (*p* < 0.001). Similarly, older adults reported lower false alarms to compound-new noncompound words than to noncompound-new compound words (*p* < 0.001), but the false alarms between the compound-new compound and noncompound-new noncompound words was not different (*p* = 0.95).

**Table C1**

Mean proportions of Hits and FAs across level of unitization, groups and retrieval conditions for behavioral experiment (M±SD)

| Groups and level of unitization  Associative test | Younger adults | | Older adults | |
| --- | --- | --- | --- | --- |
|  | compound words | noncompound words | Compound words | noncompound words |
| Hits to intact pairs | 0.85(0.08) | 0.69(0.15) | 0.85(0.11) | 0.65(0.17) |
| FAs to new compound | cc0.35(0.18) | nc0.34(0.24) | 0.33(0.22) | 0.32(0.22) |
| FAs to new noncompound | cn0.16(0.14) | nn0.22(0.18) | 0.18(0.14) | 0.32(0.23) |

Appendices D

Schematic illustration of pre-experimental and experimental familiarity, as well as association strength within the word pairs

| Retrieval pairs | Pre-experimental familiarity | Experimental familiarity | Association strength |
| --- | --- | --- | --- |
| Compound-intact (e.g., Religious Belief) | + | + | + |
| Compound-new compound (e.g., Greek Alphabet) | + | - | - |
| Compound-new noncompound (e.g., English Mythology) | - | - | - |
| Noncompound-intact (e.g., River Count) | - | + | - |
| Noncompound-new noncompound (e.g., Atmosphere Package) | - | - | - |
| Noncompound-new compound (e.g., Influence Factor) | + | - | - |
